# Potential Current and Future Distribution of the Long-Whiskered Owlet (*Xenoglaux loweryi*) in Amazonas and San Martin, NW Peru

**DOI:** 10.3390/ani12141794

**Published:** 2022-07-13

**Authors:** Gerson Meza Mori, Nilton B. Rojas-Briceño, Alexander Cotrina Sánchez, Manuel Oliva-Cruz, Christian M. Olivera Tarifeño, Marlon Y. Hoyos Cerna, Jhonny D. Ramos Sandoval, Cristóbal Torres Guzmán

**Affiliations:** 1 Instituto de Investigación para el Desarrollo Sustentable de Ceja de Selva (INDES-CES), Universidad Nacional Toribio Rodríguez de Mendoza de Amazonas (UNTRM), Chachapoyas 01001, Peru; nrojas@indes-ces.edu.pe (N.B.R.-B.); alexander.cotrina@untrm.edu.pe (A.C.S.); soliva@indes-ces.edu.pe (M.O.-C.); cristobal.torres@untrm.edu.pe (C.T.G.); 2 Department for Innovation in Biological, Agri-Food and Forest Systems, Università degli Studi della Tuscia, Via San Camillo de Lellis, 4, 01100 Viterbo, Italy; 3 Servicio Nacional de Áreas Naturales Protegidas por el Estado (SERNANP), Bagua Grande 01621, Peru; colivera@sernanp.gob.pe (C.M.O.T.); mhoyos@sernanp.gob.pe (M.Y.H.C.); jramoss@sernanp.gob.pe (J.D.R.S.)

**Keywords:** conservation, deforestation, MaxEnt, protected areas, species distribution models (SDM)

## Abstract

**Simple Summary:**

The long-whiskered owlet (*Xenoglaux loweryi*) is threatened by human activities and is currently listed as vulnerable by the IUCN. Here, we geo-referenced long-whiskered owlet records, identified key environmental variables affecting their distribution, and predicted their current and future distribution (2050 and 2070) in the Amazonas and San Martin areas of northwestern Peru. Under current conditions, areas with “high”, “moderate”, and “low” probability for the distribution of *X. loweryi* cover about 0.16% (140.85 km^2^), 0.46% (416.88 km^2^), and 1.16% (1048.79 km^2^) of the study area, respectively. Moreover, under future conditions, the “high”, “moderate”, and “low” probability areas showed profits and losses in terms of habitat suitability. Importantly, the natural protected areas in Amazonas and San Martin, both in current and in the future conditions, do not cover most of the pivotal habitats for *X. loweryi*. Furthermore, it was evident that the combination of climate change and anthropogenic activities will lead to further habitat loss for this species. Therefore, to effectively conserve this species over time, it is strongly recommended that areas with “high” (and even “moderate”) probability and the main ecosystems that this species inhabits be designated as priority areas for research and conservation (including in natural protected areas).

**Abstract:**

The IUCN has listed the long-whiskered owlet (*Xenoglaux loweryi*) as vulnerable due to the presence of few geographic records, its restricted range, and anthropogenic threats. Its natural history and ecology are largely unknown, and its distribution is widely debated; therefore, there is an urgent need for the real-time conservation of *X. loweryi*. In this study, 66 geo-referenced records of *X. loweryi*, 18 environmental variables, and the maximum entropy model (MaxEnt) have been used to predict the current and future (2050 and 2070) potential distribution of *X. loweryi* in the Amazonas and San Martin regions of northwestern Peru. In fact, under current conditions, areas of “high”, “moderate”, and “low” potential habitat suitability cover 0.16% (140.85 km^2^), 0.46% (416.88 km^2^), and 1.16% (1048.79 km^2^) of the study area, respectively. Moreover, under future conditions, the “high”, “moderate”, and “low” probability areas present profits and losses in terms of habitat suitability. Based on the environmental variables, this species mostly inhabits areas with a forest fraction with presence of trees with an emergent tree canopy of ~10–30 metres and depends on Yunga montane forest habitats with high humidity but it is not dependent on bare cover area, crops, or grasslands. Nevertheless, most of the current and future distribution areas are not part of the protected natural areas of Amazonas and San Martin. Additionally, the combination of climate change and anthropogenic activities contribute to further losses of this species habitat. Therefore, from the management point of view, corrective and preventive actions will help to preserve this species over time.

## 1. Introduction

The neotropical long-whiskered owlet (*Xenoglaux loweryi*) [1] is a Peruvian endemic bird with a restricted range [2,3]. This species inhabits the humid montane forests of the eastern cordillera of the Andes, with unique records in the departments of Amazonas and San Martin, both of which are located northeastern Peru [1,4,5,6]. The long-whiskered owlet mainly feeds on insects [6], and it is currently estimated that the total population comprises 250 to 999 individuals [7]. The behaviour and life history of *X. loweryi* are still largely unknown [4]. This may, in part, be due to the fact that they are small nocturnal owls (13–14 cm) and that their habitat is difficult to access [1,2,7]. Although only the range has been recorded for this species [2,5,8], information on their ecology and biology is still deficient, and its interactions with other species remain more or less unknown [1,7]. In their first report, O’Neil and Graves mentioned that *Xenoglaux* genus is closely related to *Glaucidium* and *Micrathene*, which share some similar characteristics and belong to the family Strigidae [1]. Owls such as the white-throated screech-owl (*Megascops albogularis*) [9], cinnamon screech-owl (*Megascops petersoni*) [10], and band-bellied owl (*Pulsatrix melanota*) [11] also cover the *Xenoglaux’s* habitat and have a greater distribution range and have been classified as species of least concern (LC) by the International Union for Conservation of Nature (IUCN); however, there is an interaction and the possibility to coexist between the aforementioned owls. Nevertheless, *X. loweryi* has been individually studied in Yunga montane forests in photo-graphic censuses, with the aim to locate this species or to obtain new records; however, according to the literature reviewed, there is such a scientific gap, e.g., intra-guild competition and/or predation, between species in this area of Peru [12,13]. 

On the other hand, there is detailed information that anthropogenic threats, such as the opening of roads, deforestation, forest fires, migratory agriculture, cattle ranching, concessions, and mining activities, are gradually reducing this species’ habitat [14,15,16,17]. At the same time, negative changes in these landscapes can significantly reduce owl populations [18,19,20]. As a result, the IUCN list *X. loweryi* as vulnerable (VU) [21], and the red book of the threatened wild fauna of Peru has listed it as endangered (EN) [14]. 

Notwithstanding, studies have shown that climate change could alter the structure and functions of the natural ecosystem that serves as a habitat for fauna species worldwide [12,22,23]. Therefore, any alteration in the climatic variables could have a negative influence on species with restricted suitable habitats [23,24]. The impact that climate change will have on the biodiversity of the Yunga montane forests, home to endemic species of both fauna and flora, and particularly on *X. loweryi* in northwest Peru, is largely unknown. However, there is a similar study about the distribution of spectacled bear (*T. ornatus*) that includes these habitats under current and future conditions in the face of climate change [25]. As for owls, studies have been carried out internationally on their current distribution [26,27], interactions with other species, feeding, reproductive success, and climate change [12,13]. Locally, there have been few studies on birds that are highly vulnerable to climate change, and this gap in knowledge means that they are not considered to be threatened with extinction [24]. Consequently, the systematic and regular collection of information on the current and future ecology and distribution of these species is an urgent priority [24]. 

In that sense, using a maximum entropy modelling approach (MaxEnt) [28], it is possible to identify the relationship between a species and its habitat by modelling its potential distribution in geographic spaces with similar characteristics to those required by the geo-referenced occurrence data [29]. MaxEnt (https://biodiversityinformatics.amnh.org/open_source/maxent/; accessed 27 May 2020) is an easy-to-use software [30], and has effectively been used in modelling rare species with narrow ranges and using only sparse occurrence data [28,31]. In addition, this biogeographic distribution model has been used extensively in studies of endangered birds, protected area planning, climate change impacts, and influences on bird distribution [23,27,32,33,34,35,36].

Therefore, this study modelled the potential current and future distribution of *X. loweryi* in the Amazonas and San Martin areas of northwestern Peru, explaining the species–habitat relationship through the response curves derived from the final environmental variables. In this vein, the objective is not only to determine the current and future distribution, but also to determine the response of the variables used for the model and an up-to-date quantitative explanation of the descriptive concepts of the habitat of *X. loweryi* (for example, whether the species inhabits humid montane forests). Suitable area comparisons were also made, including those using current and future maps, the IUCN map, and the maps from previous studies to find suitable areas in common across time. Furthermore, climate change was combined with anthropogenic disturbances, e.g., urban areas, access to road networks, among others, that are frequently found in the study area. Finally, the final distribution maps were superimposed with the different categories of natural protected areas (NPA), thus determining if the geographic extensions of NPAs satisfactorily cover this species’ habitat for protection purposes. 

Accordingly, we (i) constructed a base of georeferenced occurrence records of the species and (ii) identified and selected important and uncorrelated environmental variables, with which we (iii) modelled maps and determined changes in the environmentally suitable area under the current conditions and futures condition by 2050 and 2070. More specifically, the following questions were addressed: (1) Is there a relationship between the final environmental variables and species habitat? (2) Will the current habitat decrease under future conditions by 2050 and 2070? (3) Will the range of the predicted current distribution be larger compared to the IUCN map? (4) Will the combination of climate change and human disturbance further limit the species’ habitat suitability? (5) Will the different categories of natural protected areas cover significant areas of the current and future distribution to protect the species?

## 2. Materials and Methods

### 2.1. Study Area

**Figure 1:**
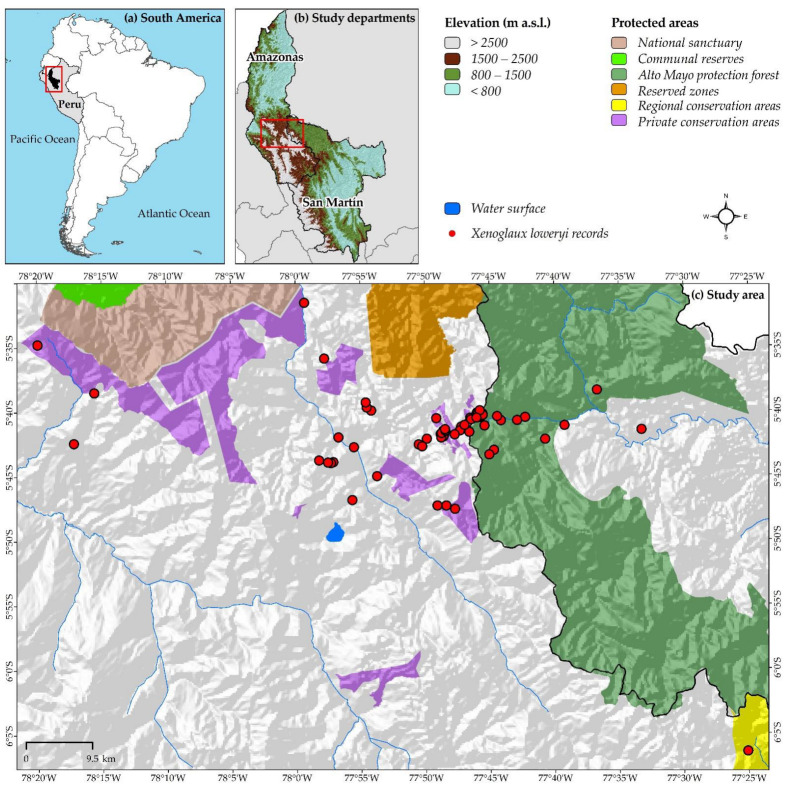
(**a**) South America; (**b**) Study departments; (**c**) Geographic location and protected areas in Amazonas and San Martin (Peru). Natural protected area categories of Amazonas and San Martin: NP: national park, NS: national sanctuary, CR: communal reserve, AMPF: Alto Mayo protected forest, RZ: reserved zones, RCA: regional conservation areas, PCA: private conservation areas.

**Figure 2:**
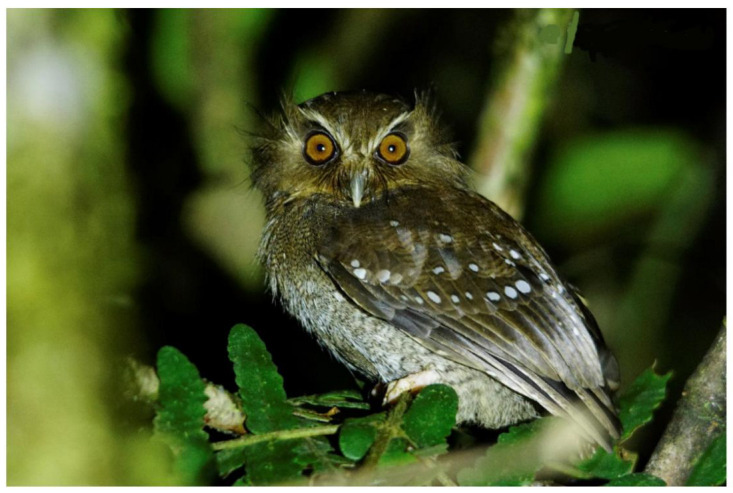
*X. loweryi*’s natural habitat in montane forests of Fundo Alto Nieva—San Martin. Photo taken by Kenny Neill Rodriguez Añazco.

The study covers the departments of Amazonas and San Martin in northwestern Peru and are located between the parallels 3°0′ and 9°0′ south and the meridians 75°50′ and 78°80′ west (Figure 1). These are unique territories where records of *X. loweryi* have been reported (Figure 2) [1,4,5]. The department of Amazonas covers an estimated area of 42,050.32 km^2^ of rugged landscape and is largely under the Amazonian rainforest. This department is characterized by a “hot and humid”, “hot dry”, and “warm temperate and slightly humid” climate, with maximum temperatures of 40 °C in the lowland forest to the north and minimum temperatures of 2 °C in the highlands to the south. In some areas, there is a water shortage of 924 mm/year, while in others, there is a surplus of up to 3000 mm/year [37]. On the southern border, the department of San Martin has an estimated area of 48,307.81 km^2^ and is located in the Peruvian Amazon between the natural high-jungle and low-jungle regions. Due to its geographical location, San Martin has a warm, hot, and humid climate. The average annual temperature is 24 °C. The maximum rainfall is 280 mm from October to March, and the region experiences small precipitation events of more than 50 mm during July and August [38,39].

### 2.2. Georeferenced Records of X. loweryi

Georeferenced (latitude/longitude) records of sightings were used and were obtained from (i) the Global Biodiversity Information Facility (GBIF) (https://www.gbif.org/; accessed on 20 July 2020), a worldwide virtual database, using QGIS Plugin version 3.12 (GBIF occurrences) (https://qgis.org/es/site/forusers/download.html; accessed on 2 March 2020) [40]; (ii) from the literature [5], and (iii) from sightings from park rangers of the National Service of Natural Protected Areas by the State of Peru (SERNANP). Subsequently, duplicate data (same longitude and latitude values) were removed [41]. Finally, all presence points went through a selection process to avoid oversampling intensity and to improve model performance [42]. Therefore, the records were filtered to a 50 m grid, reducing 431 georeferenced records to 66 records (Figure 1).

### 2.3. Environmental Variables

In this study, 32 environmental variables (Table S1) were used, including 19 bio-climatic variables and 3 topographic variables (altitude, slope, and aspect) to represent the availability of shelter to the species (tree cover, shrubs, grass, crops, bare cover, ecosystems, and tree height), proximity to water sources, and other environmental conditions (relative humidity and solar radiation). It has been shown that the use of habitat variables not overestimate the model when used for endemic birds [43]. Bioclimatic variables were obtained at a spatial resolution of 30 arcseconds (~1 km) from the global climate geodatabase, WorldClim (http://worldclim.org (accessed on 1 November 2020)). Version 2.1 [44] was used for current conditions (average 1970–2000), and version 1.4 [45] was used for the periods of 2050 (average 2041–2060) and 2070 (average 2061–2080). For 2050 and 2070, we considered greenhouse gas emission scenarios (RCP 4.5 and 8.5) based on the Representative Concentration Pathways (RCPs) [46] of the Community Climate System Model ver. 4 (CCSM4) [47,48]. Specifically, the first intermediate mode-radiated emission scenario (RCP 4.5) and the high-emission scenario (RCP 8.5) were considered [23,49]

The topographic variables were derived from the 250 m spatial resolution Digital El-evation Model (DEM) downloaded from the CGIAR Consortium for Spatial Information portal (http://srtm.csi.cgiar.org/ (accessed on 2 June 2020)). This DEM was generated from Shuttle Radar Topography Mission (SRTM) data [50]. Proximity to water sources was generated using the Euclidean distance algorithm with a spatial resolution of 250 m. The vector layer of the hydrography (rivers and lakes at a scale of 1: 100,000) of the quadrants covering Amazonas and San Martin from the National Chart of the National Geographic Institute (IGN) of Peru was used and was obtained from the website of the Ministry of Education [51]. For shelter availability, the cover probability layers were obtained from Copernicus Global Land Service LC100-V2.0 [52], the ecosystem layer was obtained from the Ministry of Environment (MINAM) [53], and tree height was determined from Global Forest Canopy Mapping [54]. Solar radiation was also obtained from WorldClim 2.1 (http://worldclim.org (accessed on 1 November 2020)) and relative humidity was obtained from the Climate Research Unit (CRU) surface climate dataset [55]. The latter was interpolated using the ordinary kriging method in ArcGIS ver 10.5 (Esri, Redlands, California, USA), with semivariogram models (gaussian, spherical and exponential) [56] yielding the best-performing spherical model, with a Root Mean Squared Error (RMSE) of 0.52 and a coefficient of determination (R^2^) of 0.98.

Variables were resampled at a 100 m resolution with QGIS version 3.12. All of the variables were then converted to ASCII format for use in the model. Additionally, non-bioclimatic variables were assumed to be unchanged for 2050 and 2070 [23,33].

### 2.4. Selection of Environmental Variables

**Figure 3:**
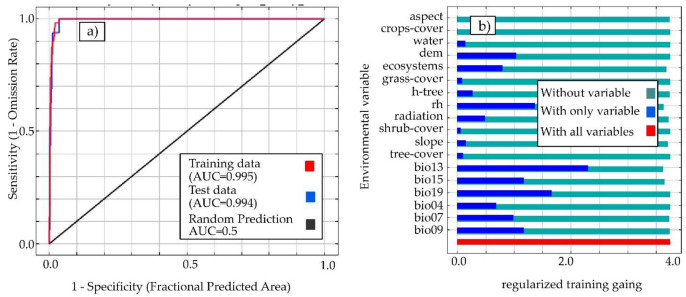
(**a**) ROC curve from the MaxEnt model. (**b**) The Jackknife test indicates the contribution of different environmental variables to the species’ current distribution.

**Figure 4:**
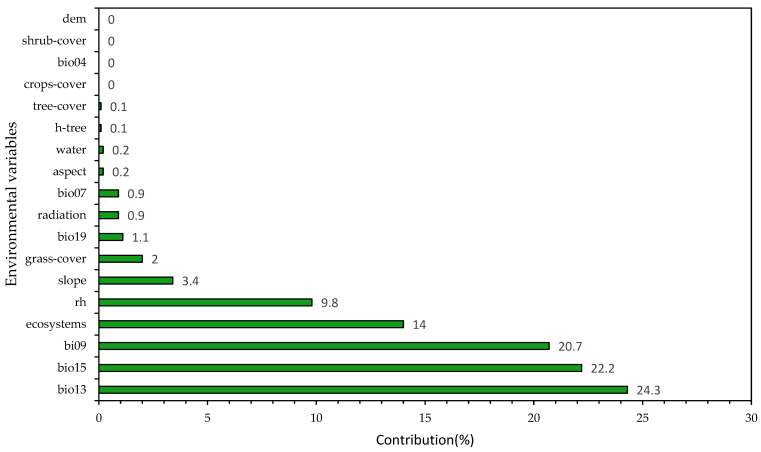
Contribution of environmental variables to the performance of the MaxEnt model.

The variables for species distribution models are very important because if all of them are incorporated into the model, some will be biologically significant while others will be of little importance [57]. However, to minimize the collinearity of the environmental variables, we used the R programming language and calculated Pearson’s correlation coefficients (| r | > 0.7) for 31 environmental variables (Table S2), an appropriate indicator in case collinearity begins to distort the model estimation [58]; however, the ecosystems were not correlated by logical inference because they were not collinear as the bioclimatic variables were, but rather were categorical variables. When two variables were highly correlated, the variable with the highest ecological importance was retained and considered to be the variable from a preliminary model of the species making the largest contribution according to Jackknife (Figure S1 and Figure 3), eliminating the second variable, thus obtaining a subset of uncorrelated variables. This resulted in a subset of 18 minimally correlated variables (Figure 4).

### 2.5. Modelling under Current and Future Climate Change Scenarios

Maxent 3.4.1 (https://biodiversityinformatics.amnh.org/open_source/maxent/; accessed on 27 May 2020) [28] was used to model the habitat suitability of *X. loweryi* in the study area. A total of 75% and 25% of the georeferenced records (randomly selected) were used to train and validate each model, respectively. The algorithm was run using 10 replicates in 5000 iterations with different random partitions (Bootstrap method) with a convergence threshold of 0.000001 and 10,000 maximum background points [59]. Additionally, the Jackknife method was used to measure the importance of the variables in habitat mapping. The area under the curve (AUC) obtained from a receiver operating characteristic (ROC) curve was used to evaluate our model [28,60]. The area under the ROC function (AUC) is a performance index that is widely used to indicate model stability to discriminate between suitable and unsuitable habitats [61]. In general, the AUC should be between 0.5 and 1: when the AUC is equal to 0.5, model performance is equivalent to the pure guess; therefore, model performance was categorized as failed (0.5–0.6), poor (0.6–0.7), fair (0.7–0.8), good (0.8–0.9), and excellent (0.9–1) [28,62,63]. A logistic output which is interpreted as the probability of the presence of a species given the environmental variables, was chosen [30]. This resulted in the total habitat suitability area of *X. loweryi* having values from 0 to 1, where 0 is an extremely unsuitable habitat, and 1 is the most suitable habitat [28,64]

Due to the restricted range characteristics of the species, thresholds were not considered, which would further restrict the species’ area. Therefore, it was reclassified into four suitable ranges: “high” (>0.6), “moderate” (0.4–0.6), and “low” (0.2–0.4) habitat suitability as well as “unsuitable habitat” (<0.2) [25,65,66].

### 2.6. Assessment of Habitat Range Change under Current, Future and IUCN Geographic Boundaries

The five reclassified maps of the potential distribution of *X. loweryi* were analyzed: one map for the current distribution, and four maps representing the future climate change scenarios for 2050 and 2070. They were overlaid on one another other to determine changes in habitat ranges. The map of the potential distribution under current conditions was also compared to the current IUCN distribution map for *X. loweryi*, which helped to validate the model. In addition, using the IUCN map as a mask, the five maps were extracted and overlaid to determine changes in the habitat ranges under these geographical boundaries.

### 2.7. Changes in Habitat Suitability under Both Climate Change and Human Disturbance Combined

Human disturbance and climate change variables were combined to assess integrated habitat impacts for *X. loweryi* following the analysis of Zhent et al. [67]. In that sense, we selected four types of human disturbances, (urban areas from the National Geographic Institute (https://www.gob.pe/ign (accessed on 18 April 2020)), roads obtained from the Ministry of Transport and Communications [68], and hydroelectric power plants and power transmission lines from GEO GPS PERU (www.geogpsperu.com accessed on 28 October 2020) to represent the most frequently occurring disturbances in the study area. Consequently, the range of influence was set at a distance of 3 km for roads, and 2 km was set as the influence area for other disturbances. The researchers’ criteria established the areas of impact. Finally, all human disturbances were integrated, and then an overlay analysis was performed on the distribution maps for 2050 and 2070 in ArcGIS ver 10.5. This resulted in maps of showing the combined influence of climate change and human disturbances on the habitat suitability for *X. loweryi* for 2050 and 2070.

### 2.8. Identifying Habitat Changes and Priority Areas for Research and Conservation

Finally, the potential distributions were overlaid with the natural protected areas (PNA) system, which was obtained from the geoserver of the National Service of Natural Areas Protected by the State—SERNANP [69], which contributed to determining the spatial distribution of the studied species within the PNAs. This enabled the identification of geographic spaces to protect the species and to identify areas with potential distribution in which research and the future establishment of NPAs will be prioritized.

## 3. Results

### 3.1. Model Performance and Contributions of Environmental Variables

The model performance obtained shows an AUC > 0.995 (Figure 3a), indicating an excellent model. The results of the Jackknife test show the importance of the different environmental variables in mapping the habitat suitability of *X. loweryi* (Figure 3b).

Five environmental variables mainly drove the model: bio13 (precipitation of wettest Month), bio15 (precipitation seasonality (coefficient of variation)), bio09 (mean temperature of driest quarter), ecosystems, and relative humidity, which contributed to 91% of the habitat predictions (Figure 4).

**Figure 5:**
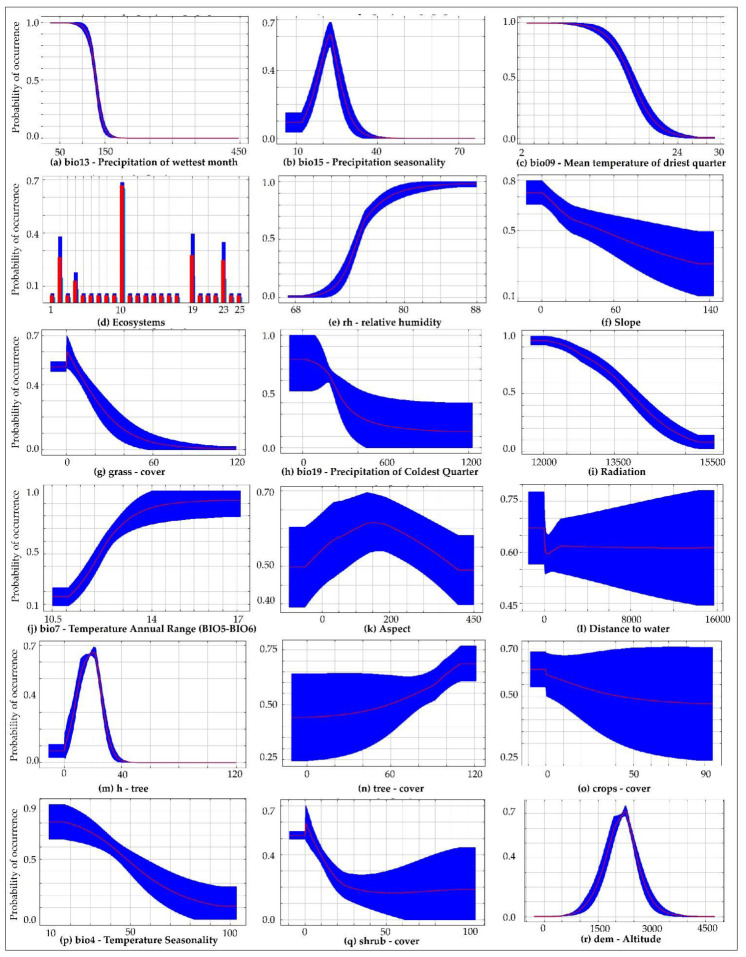
Response of environmental variables. In the figure, the y-axis indicates the probability of the species occurrence (logistic output). The red curves show the mean response, and the blue ranges are the standard deviation (±SD) calculated by 10 replicates. Most influential ecosystems for potential habitat suitability of *X. loweryi* (Figure 5d): 10, Yunga montane forest; 2, Yunga montane (pluvial) forest and 19, grassland/herbazales.

The response curves show the effects of individual variables for the prediction of the *X. loweryi* model (Figure 5). The results revealed that the response of different variables in the model was non-linear. These curves explain the response of the different variables contributing the most (Figure 5a–e) and the least (Figure 5f–r), but no less important for mapping the habitat suitability of *X. loweryi*. Under current conditions (Figure 5), the results show that *X. loweryi* depends on areas with a forest fraction with the presence of trees (Figure 5n), an emergent canopy of ~10–30 meters (Figure 5m), and Yunga montane forest habitats (Figure 5d, ecosystem type description Table S3) with high humidity (Figure 5e); meanwhile, it is not dependent on shrub (Figure 5q), grassland (Figure 5g), or cultivated areas (Figure 5o). Its habitat is not located on steep slopes (Figure 5f) and have limited elevation thresholds (Figure 5r), although they do demand proximity to water sources (Figure 5l). Moreover, solar radiation defines the microclimatic stability of the mountainous ecosystem for the species (Figure 5i).

Regarding the bioclimatic variables, the distribution of *X. loweryi* decreases in areas with precipitation higher than ~150 mm during the wettest month (Figure 5a); in theses areas, there is a probability of species occurrence in when there is a narrow range according to precipitation seasonality (20–30 mm) (Figure 5b) and with a mean temperature lower than 24 °C in the driest quarter (Figure 5c). In addition, other variables such as coldest quarter precipitation less than 600 mm (Figure 5h); an annual temperature range (BIO5–BIO6) from 11 °C to 17 °C (Figure 5j); and temperature seasonality ranging from 10 °C to 80 °C (Figure 5p) contribute to the species’ habitat conditions.

### 3.2. Potential Distribution under Current and Climate Change Scenarios of X. loweryi

Under current conditions, the distribution of *X. loweryi* in the study area covers a geographical extent of “high”, “moderate”, and “low” suitability, comprising 140.85 km^2^ (0.16%), 416.88 km^2^ (0.46%), and 1048.79 km^2^ (1.16%), respectively (Table 1). Under future conditions, the habitat shows a slight increase from the current distribution. In the “high” potential distribution area of RCP 4.5, the habitat will decrease by −10.3% in 2070, and in RCP 8.5, it will decrease by −4.9% in 2050 (Table 1). The same trend is also observed for “moderate” and “low” potential habitat suitability areas. Finally, the total area of suitability covers only 1.78% (1606.52 km^2^) of the territory in the Amazonas and San Martin regions (Table 1). In this sense, the increase in the total future area is only driven by climate change in all of the RCPs (except for RCP 4.5 in 2070), regardless of the current and future anthropogenic disturbances.

**Figure 6:**
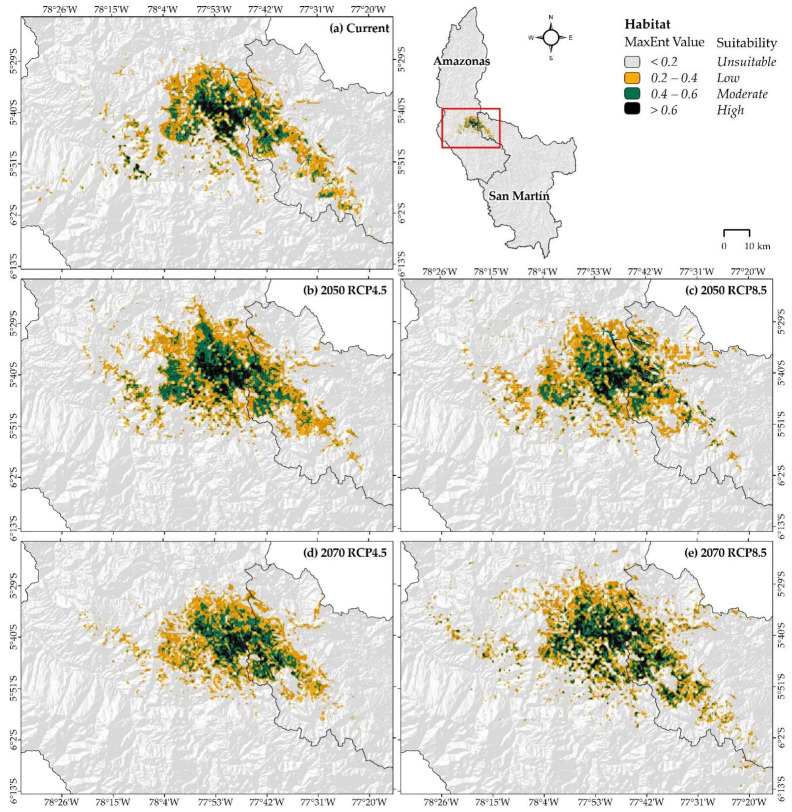
Potential current distribution of *X. loweryi* and climate change scenarios in Amazonas and San Martin, NW Peru.

In Figure 6, the current distribution (Figure 6a) and in climate change scenarios for RCP 4.5 and 8.5 in 2050 (Figure 6b–c) and 2070 (Figure 6d–e) shows the following: Currently, suitable habitats were predicted between the latitudes 5°29′0″ S and 6°2′0″ S in northwestern Peru (Figure 6a). With RCP 4.5 for 2050 (Figure 6b) and RCP 8.5 for 2070 (Figure 6e), there is an increase in the suitable habitat area for *X. lowery*. In RCP 4.5 for 2050, the increases are more concentrated, with slight gains to the south and north (Figure 6b). On the other hand, RCP 8.5 for 2070 expands both to the northwest and southeast, but the central part in this range appears to be more fragmented (Figure 6e). At RCP 8.5 in 2050 (Figure 6c) and at RCP 4.5 in 2070 (Figure 6d), there are substantial habitat losses to the southeast that shift slightly to the south and west.

The IUCN map indicates that 1.8% (1627.02 km^2^) of the study area (Amazonas and San Martin) is in the “existing” zone of occurrence for *X. loweryi* (Figure 6), while the model under current conditions predicts that 1.78% (1606.52 km^2^) is under one of the potential habitat suitability statuses (sum of “high”, “moderate”, or “low”) (Table 1). The predicted suitable areas cover 47.07% (765.88 km^2^) of the “existing” zone of the IUCN map (Table 2). When reclassifying the “existing” zone of the IUCN map into distribution ranges, it covers 86.2% (121.39 km^2^), 59.5% (248.09 km^2^), and 37.8% (396.40 km^2^) of the current “high”, “moderate”, and “low” potential habitat suitability area, respectively (Table 2). Furthermore, it is determined that the total potential habitat suitability of the IUCN map overlay, which covers 0.85% (765.88 km^2^) of the study area in future and recategorized scenarios, will generate further losses in both RCP 8.5 by 2050 and RCP 4.5 by 2070 (Table 2).

### 3.3. Changes in Habitat Suitability under Both Climate Change and Human Disturbance Combined

**Figure 7:**
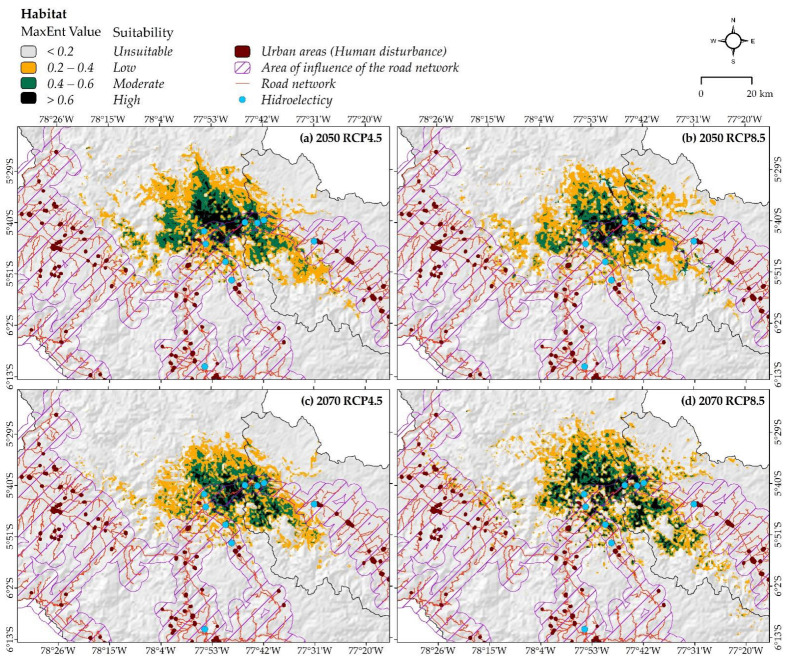
Overlapping human disturbance factors in future scenarios of *X. loweryi* in Amazonas and San Martin regions (Peru).

The habitat distribution mapping integrating climate and human disturbance shows that the areas of “high”, “moderate”, and “low” potential habitat suitability will decrease considerably (Figure 7). Specifically, the total area of suitability that will decrease significantly by 2050 is RCP 8.5 (−74.8%) and RCP 4.5 by 2070 (−76%) (Table 3) if we only consider the influence of climate change (Table 2). On the other hand, the highest habitat contraction in the areas with “high” habitat suitability will be observed in RCP 4.5: −49.7% for 2050 and −54.2% for 2070.

### 3.4. Stability and Habitat Loss of X. loweryi in Protected Areas

Only 40.5% (650.76 km^2^) of the total predicted suitable area the under current conditions in Amazonas and San Martin (1606.52 km^2^; Table 1) is covered by protected areas, the habitat suitability details of which are presented in Table S4. These results include 28.9% (40.76 km^2^), 44.3% (184.77 km^2^), and 40.5% (425.23 km^2^) coverage in the “high”, “moderate”, and “low” potential habitat suitability areas, respectively, when they were evaluated separately. The Alto Mayo protected forest covers the highest percentage of predicted suitable areas for *X. loweryi*, accounting for 26.1% (418.86 km^2^) of the NPA categories. Of the IUCN map in Amazonas and San Martin (1627.02 km^2^), only 58.8% (956.11 km^2^) was protected (Table 4), with the Alto Mayo protected forest (34.9%) being placed first, followed by private conservation areas (PCAs) (16.7%), national sanctuaries (NS) (5.0%), and regional conservation areas (RCA) (2.1%); moreover, they are the only protection and conservation categories for this species. Surprisingly, in all of the climate change scenarios, only about 30% of the total predicted protected areas cover suitable areas. Furthermore, the Alto Mayo protected forest followed by reserved zones (RZs) and PCAs cover the highest percentages of predicted suitable areas for *X. loweryi*.

The importance of each NPA category is also determined by the percentage of its area that is under the potential distribution of *X. loweryi*. Thus, the total predicted suitability under current conditions covers 23% of the total area of the Alto Mayo protected forest, 6.7% of PCAs, 2.7% of RZs, and less than 2% of the other categories (Table 4). On the other hand, the “existing” zone of the IUCN map occupies 31.2% of the Alto Mayo protected forest, 20.9% of NS, 17.3% of PCAs, and 0.8% of the RCAs. The predicted suitability in all climate change scenarios is highest to lowest. The Alto Mayo protected forest varies from (24.6–31.2%) the PCAs (6.8–10.2%) and the RZ (2.7–4.2%), while the other modalities occupy areas below 1% (except NS RCP 4.5 in 2050 and RCP 8.5 in 2070).

## 4. Discussion

This is the first study to report and estimate the future distribution of *X. loweryi* and to raise concerns about the decline of environmentally suitable areas for the species.

### 4.1. Environmental Variables in the Distribution of X. loweryi

The variables that influenced the suitability of the habitat of *X. loweryi* the most, with a contribution of 91% were: bio13 (precipitation of wettest month), bio15 (precipitation seasonality (coefficient of variation), bio09 (mean temperature of driest quarter), types of ecosystems, and relative humidity. Accordingly, studies describe this species habitat to be humid montane forests [1,2,4,5,6,70]; thus, the environmental variables respond in a quantitatively similar way according to the response curves (Figure 5). The remaining environmental variables (slope, grasslands, radiation, etc.) make minor contributions to habitat suitability (Table S5) but are no less important and were also evaluated to see their correlation with the already known habitat concepts of the target species. In this regard, the species has been sighted on gentle to steep slopes [5], similar to the results in (Figure 5f), which show a not very steep slope [5]. The altitude range is from 1890 to 2585 m, with a probability of 1900 to 2600 m, and the results of the altitude graph are similar (Figure 5r) [5]. The species has been sighted in 8–15 m canopy forests and in forests with scattered grasses due to grazing. It has also been observed both near and far from water sources. Indeed, this species is not dependent on the cover of shrubs, grassland, crops, and bare ground [2]. Physical environmental factors (air temperature, solar radiation) define specific microclimates, which may contribute to the climatic stability of the study area [71]. 

### 4.2. Current Distribution, under Future Climate Change Scenarios and Human Disturbance

Previous studies estimated the distribution of the species *X. loweryi* to be in an area of 1770.1 km^2^ [2,5] and 3016 km^2^ [8]. However, our study estimated a total current distribution of 1606.52 km^2^, which is smaller than previous studies and represents 1.78% of the study area; nevertheless, if only the “high” potential distribution habitat (140.85 km^2^) is considered, it is even less representative with regard to the study area. Moreover, the largest total habitat losses are expected to occur at RCP 4.5 in 2070 and at RCP 8.5 in 2050, with extreme losses occurring in the southeast. These losses may be increased om studies of intragremial competition and/or on the predation of *X. loweryi* with other species [12,13]. On the other hand, despite increasing the total suitability, the future climate change scenarios in RCP 4.5 in 2050 and RCP 8.5 in 2070 are more likely to be fragmented in the central part of the suitable area. The area expansion of species with very narrow niches, such as *X. loweryi*, is due to the fact that environmental conditions in combination with the habitat environment have become common [72]. However, when species conserve their range in the face of climate change, it is possible to obtain an adaptive or physiological acclimatization response, adjusting rapidly to environmental and landscape change [73]. Importantly, when considering the variable of anthropogenic activities combined with climate change, habitat loss will be −76% at most in RCP 4.5 by 2070. Clearly, based on our results, what would impact the future habitat of the species the most would be the road networks, and urban centers that are still far from the habitats of the species would have less of an impact. It has been documented that road networks contribute to deforestation in the Amazon [74,75,76], isolate wildlife populations, and generate reproductive changes [77,78,79,80]. Hence, human disturbances may limit the species range even more and may result in changes in local rainfall [5].

### 4.3. Conservation Priority Areas for X. loweryi

The total suitability distribution covers 47.07% of the existing IUCN area; further-more, 47.91% coincides with the northeastern expansion of the species described by Lane and Angulo [5]. This model shows a less suitable area for the *X. loweryi* habitat than the IUCN and Lane and Angulo map, which may be due to the different methodologies used to determine the distribution of the species. However, conservation managers should use both sets of maps for decision making. On the other hand, PNAs are one of the strategies for the conservation of fauna and flora in Peru [81], an example of which is the Alto Mayo protected forest, which has an area of 1820 km^2^ and covers the greatest range of distribution of *X. loweryi* with 26.1%. Nevertheless, there are distribution areas that are not categorized under any type of conservation. These areas are particularly susceptible to the opening of road networks, the establishment of crops, and extensive livestock farming [74,75,76,82,83]. Therefore, species distribution models (SDM) allow us to make decisions of critical importance for biodiversity conservation and are a tool for decision-making and for the management of wildlife and forest resources. In this regard, it is necessary to recommend that future protected areas should be established and used as one of the inputs to the geographical limits previsualized by an SDM for a species or species to be conserved and protected [84]. Moreover, models projected under climate change scenarios could further assist in this task because even if there are changes in the distribution of the species, sites with relatively stable distribution will be of the highest interest [25].

## 5. Conclusions

The curves derived from the final environmental variables responded to the model well and quantitatively explained the descriptive concepts of the habitat of *X. loweryi*. Areas with “high”, “moderate”, and “low” potential habitat suitability under the current conditions for *X. loweryi* were found to cover 0.16% (140.85 km^2^), 0.46% (416.88 km^2^), and 1.16% (1048.79 km^2^), respectively, of Amazonas and San Martin. The total suitability area, which covers 1.78% (1606.52 km^2^) of the study area, is expected to undergo two notable changes in the future: habitat gains and losses. Extreme losses will occur in the southeast, while habitat gains will occur in both the northwest and southeast; however, the central part of its distribution range is apparently more fragmented. In the comparison between the current suitable area of our model (1606.52 km^2^), the IUCN area (1627.02 km^2^), and the Lane and Angulo suitable study area (1770.1 km^2^), the latter had a greater total suitable habitat area. In fact, the current suitability model covers approximately ~47% of the existing IUCN and Lane and Angulo area, which supports the results of our model. Unfortunately, the combination of climate change and human disturbance would generate a greater loss of habitat for the species (−76%); therefore, human activities may represent a more latent risk for endangered species in the Amazon. 

Furthermore, when comparing the current and future suitability model with the Nat-ural Protected Areas map, unfavorably, these areas do not cover most of the key habitats for *X. loweryi*. Under current conditions, they only cover 40.5% and in climate change scenarios they are further reduced, covering only 30% of the species’ habitat. It is strongly recommended that immediate measures be taken to counter current and future anthropogenic malpractices. Likewise, it is also strongly recommended that research be carried out to determine the deforestation of the species’ habitat and surveys to be designed for the identification of new individuals and that studies be carried out on the competition and/or intraguild predation of *X. loweryi* with other species to yield more accurate results on the species’ habitat. Finally, the model provides inputs to better understand of the distribution and the habitat–species relationship in the departments of Amazonas and San Martin, providing vital information for species conservation for future conservation and management plans. 

## Figures and Tables

**Table 1:** Area (km^2^) of predicted potential distribution under current conditions and variation (%) of *X. loweryi* in Amazonas and San Martin (Peru) under climate change scenarios.

Habitat Suitability	Current (km^2^)	2050 ^1^		2070 (%) ^1, 2^	
		RCP 4.5	RCP 8.5	RCP 4.5	RCP 8.5
High	140.85	186.32	133.95	126.28	234.19
		*32.3*	*−4.9*	*−10.3 (−32.2)*	*66.3 (74.8)*
Moderate	416.88	553.93	461.82	404.81	529.89
		*32.9*	*10.8*	*−2.9 (−26.9)*	*27.1 (14.7)*
Low	1048.79	1216.11	1218.45	990.19	1184.01
		*16.0*	*16.2*	*−5.6 (−18.6)*	*12.9 (−2.8)*
Total	1606.52	1956.36	1814.22	1521.28	1948.09
		*21.8*	*12.9*	*−5.3 (−22.2)*	*21.3 (7.4)*

^1^ The area in km^2^ is in normal font, and the variation (%) from current conditions is in italics. ^2^ In brackets, variation (%) compared to the same RCP in 2050.

**Table 2:** Area (km^2^) of the “existing” IUCN map, which matches the predicted potential distribution under current conditions and variation (%) under climate change scenarios of *X. loweryi* in Amazonas and San Martin regions (Peru).

Habitat Suitability	Current IUCN (km^2^)	2050 ^1^		2070 (%) ^1, 2^	
		RCP 4.5	RCP 8.5	RCP 4.5	RCP 8.5
High	121.39	139.73	92.41	111.89	173.13
		*15.1*	*−23.9*	*−7.8 (−19.9)*	*42.6 (87.3)*
Moderate	248.09	305.20	253.31	250.25	289.86
		*23.0*	*2.1*	*0.9 (−18.0)*	*16.8 (14.4)*
Low	396.40	422.30	437.45	433.38	396.28
		*6.5*	*10.4*	*9.3 (2.6)*	*0.0 (−9.4)*
Total	765.88	867.22	783.18	795.52	859.27
		*13.2*	*2.3*	*3.9 (−8.3)*	*12.2 (9.7)*

^1^ The area in km^2^ in normal font, and the variation (%) from current conditions in italics. ^2^ In brackets, variation (%) compared to the same RCP in 2050.

**Table 3:** Changes in habitat suitability of *X. loweryi* in the study area considering human disturbance factors by 2050 and 2070.

Habitat suitability	Current (km^2^)	2050 ^1^		2070 (%) ^1, 2^	
		RCP 4.5	RCP 8.5	RCP 4.5	RCP 8.5
High	140.85	70.78	77.02	64.54	72.43
		*−49.7*	*−45.3*	*−54.2 (* *−8.8)*	*−48.6 (−6.0)*
Moderate	416.88	112.77	108.38	108.30	121.63
		*−72.9*	*−74.0*	*−74.0 (−4.0)*	*−70.8 (12.2)*
Low	1048.79	244.51	219.47	213.22	248.08
		*−76.7*	*−79.1*	*−79.7 (−12.8)*	*−76.3 (13.0)*
Total	1606.52	428.06	404.87	386.06	442.14
		*−73.4*	*−74.8*	*−76.0 (−9.8)*	*−72.5 (9.2)*

^1^ The area in km^2^ is in normal font, and the variation (%) from current conditions is in italics. ^2^ In brackets, variation (%) compared to the same RCP in 2050.

**Table 4:** Area (in km^2^ and %) of the total predicted potential distribution under both current conditions and climate change scenarios of *X. loweryi* in natural protected area categories of Amazonas and San Martin (Peru).

NPA Modalities	IUCN	Current (km^2^)	2050 ^1^		2070 ^1^	
			RCP 4.5	RCP 8.5	RCP 4.5	RCP 8.5
National Park	0.00	0.00	0.00	0.00	0.00	0.00
	*0 (0)*	*0 (0)*	*0 (0)*	*0 (0)*	*0 (0)*	*0 (0)*
National Sanctuary	82.12	6.39	52.92	5.73	1.87	35.62
	*5 (20.9)*	*0.4 (1.6)*	*2.7 (13.5)*	*0.3 (1.5)*	*0.1 (0.5)*	*1.8 (9.1)*
Communal Reserve	0.00	2.79	4.72	1.15	0.01	3.15
	*0 (0)*	*0.2 (0.2)*	*0.2 (0.4)*	*0.1 (0.1)*	*0 (0)*	*0.2 (0.3)*
Alto Mayo protected forest	568.20	418.86	447.51	517.98	423.09	567.15
	*34.9 (31.2)*	*26.1 (23)*	*22.9 (24.6)*	*28.6 (28.5)*	*27.8 (23.2)*	*29.1 (31.2)*
Reserved Zones	0.10	117.19	174.33	164.66	117.31	180.58
	*0 (0)*	*7.3 (2.7)*	*8.9 (4)*	*9.1 (3.8)*	*7.7 (2.7)*	*9.3 (4.2)*
Regional Conservation Areas	33.97	0.14	0.00	0.00	0.01	4.04
	*2.1 (0.8)*	*0 (0)*	*0 (0)*	*0 (0)*	*0 (0)*	*0.2 (0.1)*
Private Conservation Areas	271.72	105.39	159.84	120.46	107.45	124.91
	*16.7 (17.3)*	*6.6 (6.7)*	*8.2 (10.2)*	*6.6 (7.7)*	*7.1 (6.8)*	*6.4 (7.9)*
Total	956.11	650.76	839.31	809.99	649.74	915.44
	*58.8 (3.1)*	*40.5 (2.1)*	*42.9 (2.8)*	*44.6 (2.7)*	*42.7 (2.1)*	*47 (3)*

^1^ Area in km^2^ is in normal font and meaning is in percentage (%) in italics. No brackets indicate the percentage (%) of the area of the potential habitat range, and brackets indicate the percentage (%) of the area of the NPA modalities.

## Data Availability

The data used to support the findings of this study are available from the corresponding author upon request.

## Supplementary Materials

The following are available online at https://www.mdpi.com/article/10.3390/ani12141794/s1, Table S1: Variables for the MaxEnt model of *X. loweryi* in Amazonas and San Martin (Peru), Table S2: Pearson correlation coefficients (r) between environmental variables for modelling the potential distribution of *X. loweryi* in Amazonas and San Martin (Peru), Figure S1: Jackknife test in a preliminary model generated using only the 32 environmental variables for the modelling of the potential distribution of *X. loweryi* in Amazonas and San Martin (Peru), Table S3: A description of the ecosystem types comprising Amazonas and San Martin (Peru), Table S4: Area (in km^2^ and %) of the total potential distribution expected under both current conditions and climate change scenarios of *X. loweryi* in the different categories of natural protected areas in Amazonas and San Martin (Peru), Table S5: Contributions (%) of the environmental variables in the current suitability habitat and in the climate change scenarios.
