# Supplementary material for: Potential Current and Future Distribution of the Long-Whiskered Owlet (Xenoglaux loweryi) in Amazonas and San Martin, NW Peru
Source: Animals (Basel). 2022 Jul 13;12(14):1794. doi: 10.3390/ani12141794 (PMC9312142; doi:10.3390/ani12141794)
Supplement: Supplementary file 1 [file animals-12-01794-s001.zip › animals-1639658-supplementary.pdf]

Table S1. Variables for the MaxEnt model of *X. loweryi* in Amazonas and San Martin areas (Peru).

Category	code	Description	Unit
	Bio01	Annual Mean Temperature	°C
	Bio02	Mean Diurnal Range (mean of monthly ($T°_{máx.}$ - $T°_{min.}$))	°C
	Bio03	Isothermality (Bio02/Bio07) (* 100)	
	Bio04	Temperature Seasonality (standard deviation *100)	°C
	Bio05	Max Temperature of Warmest Month	°C
	Bio06	Min Temperature of Coldest Month	°C
	Bio07	Temperature Annual Range (Bio05-Bio06)	°C
	Bio08	Mean Temperature of Wettest Quarter	°C
	Bio09	Mean Temperature of Driest Quarter	°C
**Bioclimatic**	Bio10	Mean Temperature of Warmest Quarter	°C
	Bio11	Mean Temperature of Coldest Quarter	°C
	Bio12	Annual Precipitation	mm
	Bio13	Precipitation of Wettest Month	mm
	Bio14	Precipitation of Driest Month	mm
	Bio15	Precipitation Seasonality (coefficient of variation)	mm
	Bio16	Precipitation of Wettest Quarter	mm
	Bio17	Precipitation of Driest Quarter	mm
	Bio18	Precipitation of Warmest Quarter	mm
	Bio19	Precipitation of Coldest Quarter	mm
	alt	altitude	msnm
**Topographic**	slo	slope	%
	asp	aspect	°
	tree	tree cover	%
	shrub	shrub cover	%
	grass	grass cover	%
**shelter availability**	crop	crop cover	%
	bare	bare cover	%
	ecosystem	ecosystems	-
	th	tree height	m
	rh	relative humidity	%
**environmental variables**	srad	solar radiation	MJ m-2 day-1
	wa	proximity to water	m

Table S2. Pearson correlation coefficients (r) between environmental variables for modelling the potential distribution of *X. loweryi* in Amazonas and San Martin areas (Peru).

	bio_1	bio_2	bio_3	bio_4	bio_5	bio_6	bio_7	bio_8	bio_9	bio_10	bio_11	bio_12	bio_13	bio_14	bio_15	bio_16	bio_17	bio_18	bio_19	bare	crops	shrub	grass	tree	h_tree	dem	slope	aspect	h.relative	radiation	d_water
bio_1	1	0.74	0.96	-0.67	0.99	0.99	0.30	0.99	0.99	0.99	0.99	-0.14	-0.48	-0.30	-0.34	-0.26	-0.27	0.15	-0.22	0.03	0.06	-0.13	-0.01	0.06	0.29	-0.99	-0.03	-0.17	0.22	0.39	-0.29
bio_2	0.74	1	0.69	-0.22	0.78	0.69	0.85	0.73	0.73	0.74	0.73	-0.53	-0.56	-0.67	0.21	-0.59	-0.69	-0.14	-0.65	0.27	0.32	0.00	0.29	-0.20	-0.12	-0.72	0.07	-0.02	-0.08	0.68	-0.46
bio_3	0.96	0.69	1	-0.77	0.95	0.96	0.21	0.96	0.96	0.96	0.96	-0.18	-0.46	-0.37	-0.27	-0.27	-0.33	0.12	-0.29	0.03	0.06	-0.15	-0.06	0.09	0.35	-0.95	-0.07	-0.26	0.36	0.40	-0.28
bio_4	-0.67	-0.22	-0.77	1	-0.63	-0.70	0.27	-0.67	-0.67	-0.66	-0.68	-0.06	0.26	0.02	0.48	0.03	-0.02	-0.14	-0.03	0.06	0.05	0.35	0.40	-0.38	-0.54	0.69	0.33	0.23	-0.55	0.01	0.05
bio_5	0.99	0.78	0.95	-0.63	1	0.99	0.36	0.99	0.99	0.99	0.997	-0.18	-0.51	-0.34	-0.31	-0.30	-0.31	0.12	-0.26	0.05	0.09	-0.12	0.02	0.03	0.25	-0.98	-0.02	-0.15	0.18	0.42	-0.31
bio_6	0.99	0.69	0.96	-0.70	0.99	1	0.23	0.99	0.99	0.99	0.99	-0.10	-0.47	-0.26	-0.39	-0.23	-0.22	0.16	-0.17	0.00	0.03	-0.14	-0.05	0.08	0.33	-0.98	-0.05	-0.18	0.24	0.34	-0.26
bio_7	0.30	0.85	0.21	0.27	0.36	0.23	1	0.29	0.29	0.30	0.29	-0.59	-0.43	-0.64	0.48	-0.60	-0.70	-0.28	-0.68	0.34	0.39	0.10	0.43	-0.33	-0.42	-0.29	0.14	0.16	-0.36	0.64	-0.41
bio_8	0.99	0.73	0.96	-0.67	0.99	0.99	0.29	1	0.99	0.99	0.99	-0.14	-0.49	-0.30	-0.35	-0.26	-0.27	0.14	-0.22	0.03	0.06	-0.13	-0.02	0.06	0.29	-0.99	-0.03	-0.17	0.22	0.38	-0.29
bio_9	0.99	0.73	0.96	-0.67	0.99	0.99	0.29	0.99	1	0.99	0.99	-0.13	-0.48	-0.29	-0.35	-0.25	-0.26	0.15	-0.21	0.03	0.06	-0.14	-0.02	0.06	0.30	-0.99	-0.03	-0.17	0.22	0.38	-0.29
bio_10	0.99	0.74	0.96	-0.66	0.99	0.99	0.30	0.99	0.99	1	0.99	-0.14	-0.48	-0.30	-0.34	-0.26	-0.27	0.15	-0.22	0.03	0.06	-0.13	-0.01	0.05	0.29	-0.99	-0.03	-0.17	0.22	0.39	-0.29
bio_11	0.99	0.73	0.96	-0.68	0.99	0.99	0.29	0.99	0.99	0.99	1	-0.13	-0.48	-0.30	-0.35	-0.26	-0.27	0.15	-0.21	0.03	0.06	-0.14	-0.02	0.06	0.30	-0.99	-0.04	-0.17	0.23	0.38	-0.29
bio_12	-0.14	-0.53	-0.18	-0.06	-0.18	-0.10	-0.59	-0.14	-0.13	-0.14	-0.13	1.00	0.81	0.84	-0.63	0.96	0.84	0.85	0.84	-0.20	-0.25	0.00	-0.26	0.17	0.18	0.12	0.17	-0.08	0.23	-0.45	0.05
bio_13	-0.48	-0.56	-0.46	0.26	-0.51	-0.47	-0.43	-0.49	-0.48	-0.48	-0.48	0.81	1	0.57	-0.07	0.90	0.54	0.76	0.51	-0.08	-0.13	0.10	-0.09	0.01	-0.06	0.48	0.25	-0.04	0.25	-0.25	-0.01
bio_14	-0.30	-0.67	-0.37	0.02	-0.34	-0.26	-0.64	-0.30	-0.29	-0.30	-0.30	0.84	0.57	1	-0.72	0.76	0.98	0.44	0.98	-0.26	-0.31	-0.08	-0.35	0.26	0.14	0.27	0.02	0.05	0.14	-0.56	0.30
bio_15	-0.34	0.21	-0.27	0.48	-0.31	-0.39	0.48	-0.35	-0.35	-0.34	-0.35	-0.63	-0.07	-0.72	1	-0.43	-0.76	-0.40	-0.79	0.22	0.24	0.16	0.36	-0.30	-0.39	0.37	0.08	0.09	-0.16	0.46	-0.12
bio_16	-0.26	-0.59	-0.27	0.03	-0.30	-0.23	-0.60	-0.26	-0.25	-0.26	-0.26	0.96	0.90	0.76	-0.43	1	0.75	0.84	0.75	-0.22	-0.26	-0.02	-0.24	0.17	0.15	0.25	0.21	-0.07	0.26	-0.41	0.06
bio_17	-0.27	-0.69	-0.33	-0.02	-0.31	-0.22	-0.70	-0.27	-0.26	-0.27	-0.27	0.84	0.54	0.98	-0.76	0.75	1	0.45	0.99	-0.25	-0.30	-0.03	-0.34	0.24	0.17	0.24	0.03	0.00	0.13	-0.62	0.33
bio_18	0.15	-0.14	0.12	-0.14	0.12	0.16	-0.28	0.14	0.15	0.15	0.15	0.85	0.76	0.44	-0.40	0.84	0.45	1	0.46	-0.09	-0.12	0.04	-0.10	0.04	0.16	-0.14	0.28	-0.14	0.25	-0.14	-0.26
bio_19	-0.22	-0.65	-0.29	-0.03	-0.26	-0.17	-0.68	-0.22	-0.21	-0.22	-0.21	0.84	0.51	0.98	-0.79	0.75	0.99	0.46	1	-0.26	-0.30	-0.03	-0.34	0.24	0.18	0.19	0.06	-0.01	0.11	-0.61	0.30
bare	0.03	0.27	0.03	0.06	0.05	0.00	0.34	0.03	0.03	0.03	0.03	-0.20	-0.08	-0.26	0.22	-0.22	-0.25	-0.09	-0.26	1	0.99	0.46	0.43	-0.58	-0.32	-0.03	0.10	0.09	-0.05	0.17	-0.16
crops	0.06	0.32	0.06	0.05	0.09	0.03	0.39	0.06	0.06	0.06	0.06	-0.25	-0.13	-0.31	0.24	-0.26	-0.30	-0.12	-0.30	0.99	1	0.36	0.45	-0.55	-0.32	-0.07	0.07	0.12	-0.06	0.19	-0.15
shrub	-0.13	0.00	-0.15	0.35	-0.12	-0.14	0.10	-0.13	-0.14	-0.13	-0.14	0.00	0.10	-0.08	0.16	-0.02	-0.03	0.04	-0.03	0.46	0.36	1	0.69	-0.90	-0.47	0.19	0.55	-0.04	-0.19	0.04	-0.20
grass	-0.01	0.29	-0.06	0.40	0.02	-0.05	0.43	-0.02	-0.02	-0.01	-0.02	-0.26	-0.09	-0.35	0.36	-0.24	-0.34	-0.10	-0.34	0.43	0.45	0.69	1	-0.93	-0.59	0.05	0.39	0.16	-0.28	0.16	-0.25
tree	0.06	-0.20	0.09	-0.38	0.03	0.08	-0.33	0.06	0.06	0.05	0.06	0.17	0.01	0.26	-0.30	0.17	0.24	0.04	0.24	-0.58	-0.55	-0.90	-0.93	1	0.59	-0.10	-0.48	-0.08	0.23	-0.14	0.25
h_tree	0.29	-0.12	0.35	-0.54	0.25	0.33	-0.42	0.29	0.30	0.29	0.30	0.18	-0.06	0.14	-0.39	0.15	0.17	0.16	0.18	-0.32	-0.32	-0.47	-0.59	0.59	1	-0.29	-0.37	0.07	0.35	-0.10	0.16
dem	-0.99	-0.72	-0.95	0.69	-0.98	-0.98	-0.29	-0.99	-0.99	-0.99	-0.99	0.12	0.48	0.27	0.37	0.25	0.24	-0.14	0.19	-0.03	-0.07	0.19	0.05	-0.10	-0.29	1	0.07	0.14	-0.23	-0.36	0.26
slope	-0.03	0.07	-0.07	0.33	-0.02	-0.05	0.14	-0.03	-0.03	-0.03	-0.04	0.17	0.25	0.02	0.08	0.21	0.03	0.28	0.06	0.10	0.07	0.55	0.39	-0.48	-0.37	0.07	1	-0.19	-0.22	0.05	-0.39
aspect	-0.17	-0.02	-0.26	0.23	-0.15	-0.18	0.16	-0.17	-0.17	-0.17	-0.17	-0.08	-0.04	0.05	0.09	-0.07	0.00	-0.14	-0.01	0.09	0.12	-0.04	0.16	-0.08	0.07	0.14	-0.19	1	-0.20	-0.01	0.16
h_relative	0.22	-0.08	0.36	-0.55	0.18	0.24	-0.36	0.22	0.22	0.22	0.23	0.23	0.25	0.14	-0.16	0.26	0.13	0.25	0.11	-0.05	-0.06	-0.19	-0.28	0.23	0.35	-0.23	-0.22	-0.20	1	0.26	0.10
radiation	0.39	0.68	0.40	0.01	0.42	0.34	0.64	0.38	0.38	0.39	0.38	-0.45	-0.25	-0.56	0.46	-0.41	-0.62	-0.14	-0.61	0.17	0.19	0.04	0.16	-0.14	-0.10	-0.36	0.05	-0.01	0.26	1	-0.24
d_water	-0.29	-0.46	-0.28	0.05	-0.31	-0.26	-0.41	-0.29	-0.29	-0.29	-0.29	0.05	-0.01	0.30	-0.12	0.06	0.33	-0.26	0.30	-0.16	-0.15	-0.20	-0.25	0.25	0.16	0.26	-0.39	0.16	0.10	-0.24	1

Figure S1. Jackknife test in a preliminary model generated using only the 32 environmental variables for the modelling of the potential distribution of *X. loweryi* in Amazonas and San Martin areas (Peru). Regularized training gain without variable (green), with only variable (blue) and with all variables (red).

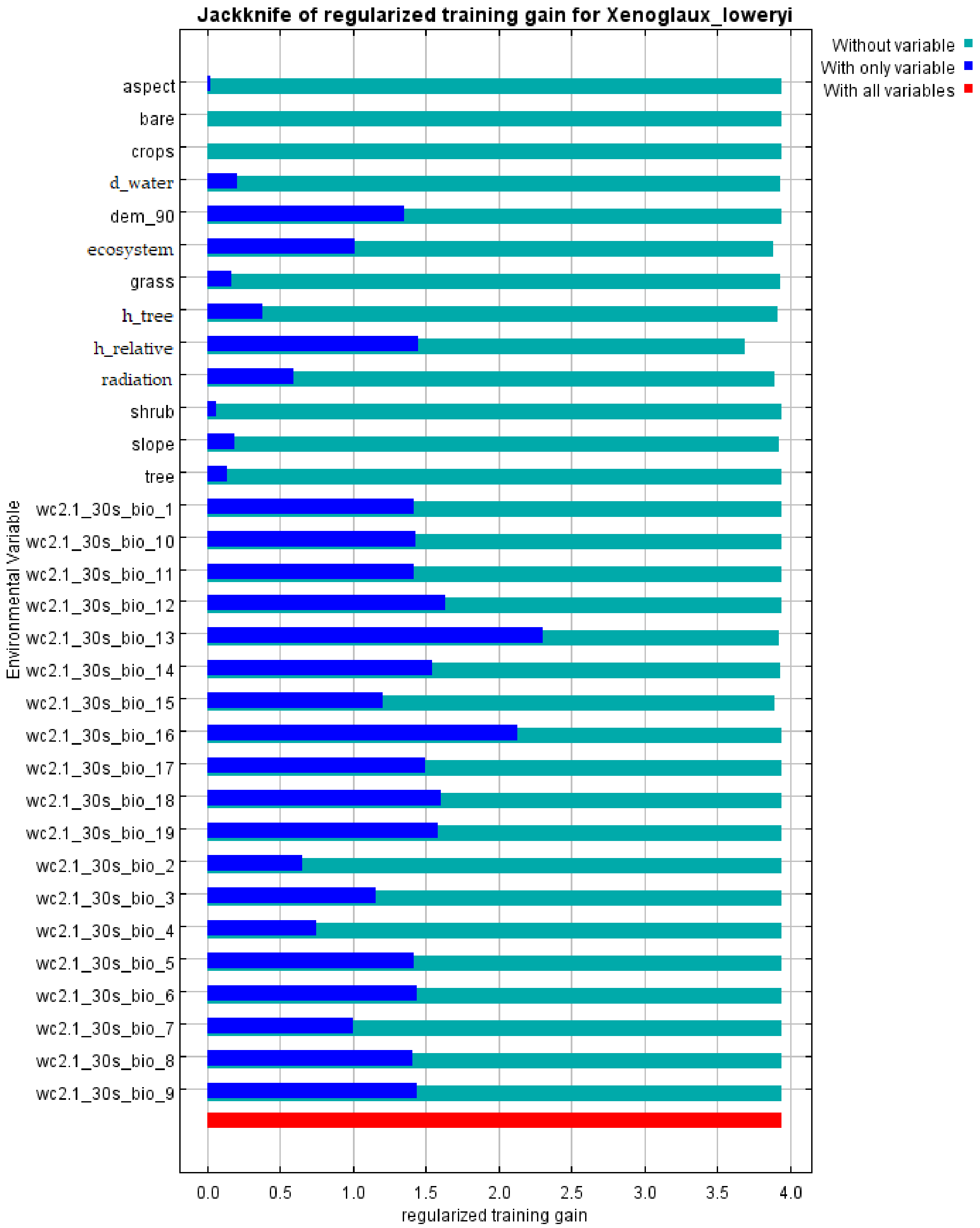

Table S3. A description of the ecosystem types comprising Amazonas and San Martin areas (Peru).

Code	description
**1**	Wetlands
**2**	Yunga high altimontane (pluvial) forest
**3**	Flooded alluvial forest
**4**	Basimontane Yunga Forest
**5**	High hill forest
**6**	Low hill forest
**7**	Non-flooded terrace forest
**8**	Inter-Andean seasonally dry forest
**9**	Seasonally dry forest in the east
**10**	Yunga montane forest
**11**	Montane relict forest on western slope
**12**	Island
**13**	Jalca region
**14**	Lake and lagoon
**15**	Andean scrubland
**16**	Puna humid grassland
**17**	Palm tree swamp
**18**	Herbaceous shrub swamp
**19**	Grasslands/Herbaceous
**20**	Plantation forestry
**21**	River
**22**	Varillales forest
**23**	Secondary forest
**24**	Agricultural area
**25**	Urban area

Table S4. Area (in km^2 and %) of the total potential distribution expected under both current conditions and climate change scenarios of *X. loweryi* in the different categories of Natural Protected Areas in Amazonas and San Martin territories (Peru).

Model	Habitat suitabilit	Area (Km^2)	Protected natural area catergories [1,2]							
			**NP**	**NS**	**CR**	**AMPF**	**RZ**	**RCA**	**PCA**	**Total**
			**17162.33**	**392.38**	**1186.05**	**1820.00**	**4347.98**	**4026.33**	**1574.63**	**30509.70**
	High	140.85	0.00	0.00	0.00	13.29	4.17	0.00	23.29	40.76
			0 (0)	*0 (0)*	*0 (0)*	*9.4 (0.7)*	*3 (0.1)*	*0 (0)*	*16.5 (1.5)*	*28.9 (0.1)*
current	Moderate	416.88	0.00	0.03	0.02	117.62	38.92	0.00	28.18	184.77
			0 (0)	*0 (0)*	*0 (0)*	*28.2 (6.5)*	*9.3 (0.9)*	*0 (0)*	*6.8 (1.8)*	*44.3 (0.6)*
	Low	1048.79	0.00	6.36	2.77	287.94	74.10	0.14	53.92	425.23
			0 (0)	*0.6 (1.6)*	*0.3 (0.2)*	*27.5 (15.8)*	*7.1 (1.7)*	*0 (0)*	*5.1 (3.4)*	*40.5 (1.4)*
	Total	1606.52	0.00	6.39	2.79	418.86	117.19	0.14	105.39	650.76
			0 (0)	*0.4 (1.6)*	*0.2 (0.2)*	*26.1 (23)*	*7.3 (2.7)*	*0 (0)*	*6.6 (6.7)*	*40.5 (2.1)*
	High	186.32	0.00	0.00	0.00	17.98	18.92	0.00	26.55	63.44
			0 (0)	*0 (0)*	*0 (0)*	*9.6 (1)*	*10.2 (0.4)*	*0 (0)*	*14.2 (1.7)*	*34.1 (0.2)*
2050	Moderate	553.93	0.00	2.62	0.00	132.33	57.30	0.00	35.49	227.73
			0 (0)	*0.5 (0.7)*	*0(0)*	*23.9 (7.3)*	*10.3 (1.3)*	*0 (0)*	*6.4 (2.3)*	*41.1 (0.7)*
RCP 4.5	Low	1216.11	0.00	50.30	4.72	297.20	98.11	0.00	97.81	548.14
			0 (0)	*4.1 (12.8)*	*0.4 (0.4)*	*24.4 (16.3)*	*8.1 (2.3)*	*0 (0)*	*8 (6.2)*	*45.1 (1.8)*
	Total	1956.36	0.00	52.92	4.72	447.51	174.33	0.00	159.84	839.31
			0 (0)	*2.7 (13.5)*	*0.2 (0.4)*	*22.9 (24.6)*	*8.9 (4)*	*0 (0)*	*8.2 (10.2)*	*42.9 (2.8)*
	High	133.95	0.00	0.00	0.00	31.66	6.18	0.00	15.47	53.31
			0 (0)	*0.0*	*0 (0)*	*23.6 (1.7)*	*4.6 (0.1)*	*0 (0)*	*11.5 (1)*	*39.8 (0.2)*
	Moderate	461.82	0.00	0.00	0.00	134.03	46.59	0.00	33.97	214.59
2050			*0 (0)*	*0 (0)*	*0 (0)*	*29 (7.4)*	*10.1 (1.1)*	*0 (0)*	*7.4 (2.2)*	*46.5 (0.7)*
RCP 8.5	Low	1218.45	0.00	5.73	1.15	352.29	111.90	0.00	71.02	542.08
			0 (0)	*0.5 (1.5)*	*0.1 (0.1)*	*28.9 (19.4)*	*9.2 (2.6)*	*0 (0)*	*5.8 (4.5)*	*44.5 (1.8)*
	Total	1814.22	0.00	5.73	1.15	517.98	164.66	0.00	120.46	809.99
			0 (0)	*0.3 (1.5)*	*0.1 (0.1)*	*28.6 (28.5)*	*9.1 (3.8)*	*0 (0)*	*6.6 (7.7)*	*44.6 (2.7)*
	High	126.28	0.00	0.00	0.00	41.87	0.88	0.00	17.96	60.72
			0 (0)	*0 (0)*	*0 (0)*	*33.2 (2.3)*	*0.7 (0)*	*0 (0)*	*14.2 (1.1)*	*48.1 (0.2)*
2070	Moderate	404.81	0.00	0.00	0.00	126.02	28.22	0.00	33.97	188.21
			0 (0)	*0 (0)*	*0 (0)*	*31.1 (6.9)*	*7 (0.6)*	*0 (0)*	*8.4 (2.2)*	*46.5 (0.6)*
RCP 4.5	Low	990.19	0.00	1.87	0.01	255.20	88.21	0.01	55.51	400.82
			0 (0)	*0.2 (0.5)*	*0 (0)*	*25.8 (14)*	*8.9 (2)*	*0 (0)*	*5.6 (3.5)*	*40.5 (1.3)*
	Total	1521.28	0.00	1.87	0.01	423.09	117.31	0.01	107.45	649.74
			0 (0)	*0.1 (0.5)*	*0 (0)*	*27.8 (23.2)*	*7.7 (2.7)*	*0 (0)*	*7.1 (6.8)*	*42.7 (2.1)*
	High	234.19	0.00	0.00	0.00	63.71	12.95	0.00	29.17	105.84
			0 (0)	*0 (0)*	*0 (0)*	*27.2 (3.5)*	*5.5 (0.3)*	*0 (0)*	*12.5 (1.9)*	*45.2 (0.3)*
2070	Moderate	529.89	0.00	0.65	0.16	159.49	54.95	0.18	29.28	244.71
			0 (0)	*0.1 (0.2)*	*0 (0)*	*30.1 (8.8)*	*10.4 (1.3)*	*0 (0)*	*5.5 (1.9)*	*46.2 (0.8)*
RCP 8.5	Low	1184.01	0.00	34.96	2.99	343.95	112.67	3.86	66.46	564.89
			0 (0)	*3 (8.9)*	*0.3 (0.3)*	*29 (18.9)*	*9.5 (2.6)*	*0.3 (0.1)*	*5.6 (4.2)*	*47.7 (1.9)*
	Total	1948.09	0.00	35.62	3.15	567.15	180.58	4.04	124.91	915.44
			0 (0)	*5 (20.9)*	*0 (0)*	*34.9 (31.2)*	*0 (0)*	*2.1 (0.8)*	*16.7 (17.3)*	*58.8 (3.1)*
IUCN	Extant	1627.02	0.00	82.12	0.00	568.20	0.10	33.97	271.72	956.11
			0 (0)	*5 (20.9)*	*0 (0)*	*34.9 (31.2)*	*0 (0)*	*2.1 (0.8)*	*16.7 (17.3)*	*58.8 (3.1)*

[1] The area in km^2 in normal font and its meaning in percentage (%) in italics. Without parentheses the percentage (%) with respect to the area of the potential habitat range and in parentheses the percentage (%) with respect to the area of the NPA modalities

[2] NP: National Park, NS: National Sanctuary, CR: Communal Reserve, AMPF: Alto Mayo protection forest, RZ: Reserved Zones, RCA: Regional Conservation Areas, PCA: Private Conservation Areas

Table S5. Contributions (%) of the environmental variables in the current suitability habitat and in the climate change scenarios.

Environmental variables	Current(%)	2050(%)		2070(%)	
		RCP 4.5	RCP 8.5	RCP 4.5	RCP 8.5
Bio13	24.3	21.4	29.9	18.7	15.2
Bio15	22.2	0.8	1	0	0.5
Bio9	20.7	27.5	14.2	26	9.5
Ecosystems	14	11.5	20.2	17.7	23
Relative humidity	9.8	3.9	7	6.1	2.6
Slope	3.4	0.6	0.5	3.3	2.3
grass cover	2	1.7	2.6	0.2	1
Bio19	1.1	20.5	21.1	24.7	19.1
radiation	0.9	3.7	1.1	1	1.5
Bio7	0.9	5.7	0	0.1	11.1
Aspect	0.2	0	0.4	0.4	0.3
D_water	0.2	0	0.1	0.1	0.1
tree height	0.1	0.4	0.2	0	0.4
Tree cover	0.1	0.3	0.4	0	0.5
Crop cover	0	0	0.4	0	0
Bio4	0	1.1	0.3	0	3.3
Shrub cover	0	0.8	0.6	1.9	1.7
Alitude	0	0.1	0.1	0	7.7
**Total**	100	100	100	100	100
